# Disease progression modeling of Alzheimer’s disease according to education level

**DOI:** 10.1038/s41598-020-73911-6

**Published:** 2020-10-08

**Authors:** Ko Woon Kim, Sook Young Woo, Seonwoo Kim, Hyemin Jang, Yeshin Kim, Soo Hyun Cho, Si Eun Kim, Seung Joo Kim, Byoung-Soo Shin, Hee Jin Kim, Duk L. Na, Sang Won Seo

**Affiliations:** 1 Department of Neurology, Samsung Medical Center, Sungkyunkwan University School of Medicine, 81 Irwon-dong, Gangnam-gu, Seoul, 06351 South Korea; 2 Department of Neurology, School of Medicine, Jeonbuk National University Hospital, Jeonju, South Korea; 3 Research Institute of Clinical Medicine of Jeonbuk National University, Jeonju, South Korea; 4 Biomedical Institute of Jeonbuk National University Hospital, Jeonju, South Korea; 5 Statistics and Data Center, Samsung Medical Center, Seoul, South Korea; 6 Neuroscience Center, Samsung Medical Center, Seoul, South Korea; 7 Department of Neurology, Kangwon National University Hospital, Kangwon National University College of Medicine, Chuncheon, South Korea; 8 Department of Neurology, Chonnam National University Medical School and Hospital, Gwangju, South Korea; 9 Department of Neurology, Inje University College of Medicine, Haeundae Paik Hospital, Busan, South Korea; 10 Department of Neurology, Gyeongsang National University School of Medicine and Gyeongsang National University Changwon Hospital, Changwon, Republic of Korea; 11 Department of Health Sciences and Technology, SAIHST, Sungkyunkwan University, Seoul, South Korea; 12 Samsung Alzheimer Research Center, Samsung Medical Center, Seoul, South Korea; 13 Department of Intelligent Precision Healthcare Convergence, Sungkyunkwan University School of Medicine, Seoul, South Korea

**Keywords:** Alzheimer's disease, Alzheimer's disease

## Abstract

To develop a disease progression model of Alzheimer’s disease (AD) that shows cognitive decline from subjective cognitive impairments (SCI) to the end stage of AD dementia (ADD) and to investigate the effect of education level on the whole disease spectrum, we enrolled 565 patients who were followed up more than three times and had a clinical dementia rating sum of boxes (CDR-SB). Three cohorts, SCI (n = 85), amnestic mild cognitive impairment (AMCI, n = 240), and ADD (n = 240), were overlapped in two consecutive cohorts (SCI and AMCI, AMCI and ADD) to construct a model of disease course, and a model with multiple single-cohorts was estimated using a mixed-effect model. To examine the effect of education level on disease progression, the disease progression model was developed with data from lower (≤ 12) and higher (> 12) education groups. Disease progression takes 274.3 months (22.9 years) to advance from 0 to 18 points using the CDR-SB. Based on our predictive equation, it takes 116.5 months to progress from SCI to AMCI and 56.2 months to progress from AMCI to ADD. The rate of CDR-SB progression was different according to education level. The lower-education group showed faster CDR-SB progression from SCI to AMCI compared to the higher-education group, and this trend disappeared from AMCI to ADD. In the present study, we developed a disease progression model of AD spectrum from SCI to the end stage of ADD. Our disease modeling provides us with more understanding of the effect of education on cognitive trajectories.

## Introduction

Understanding Alzheimer’s disease (AD) progression could be helpful in staging the current level of disease severity, establishing a management plan, predicting prognosis, and comparing the effects of treatment. However, there is limited knowledge about the temporal course of the AD spectrum, such as subjective cognitive impairments (SCI), amnestic mild cognitive impairment (AMCI), and AD dementia (ADD), because previous longitudinal studies have been conducted at a specific disease status^1–3^. Although a few studies have followed participants with normal cognition at baseline to ADD, such studies are difficult because it can take several decades to develop dementia. In addition, these ADD patients are limited in their ability to represent all ADD patients because only a small number progress to actual dementia from normal cognition even if there are a large number of participants. Therefore, a statistical modeling study is required to understand the course of disease progression in AD.

Education level has been considered an important factor influencing the course of AD progression. However, evidence relating to the effect of education on cognitive trajectories across the whole disease spectrum is controversial. That is, previous studies suggested that highly educated patients showed slower cognitive decline at the stage of normal cognition^4–6^ while highly educated patients show more rapid cognitive decline than those that are poorly educated at the dementia stage^7–9^. Therefore, it might be important to determine whether education affects the cognitive trajectory across the whole disease spectrum and whether the impact of education might vary from early stage to late stage AD.

In the present study, we estimated a model of disease progression for the whole time span over a longer period using separate multiple cohorts. Each cohort was measured longitudinally from many individuals across the disease spectrum from SCI to the end stage of ADD. We therefore aimed to develop a disease progression model of AD depending on education level that shows the effects of education level cognitive and functional performance decline from SCI to the end stage of ADD.

## Results

### Clinical characteristics of participants

Table 1 summarizes the demographic characteristics of 565 patients. The number of patients was 85, 240, and 240 in the SCI, AMCI, and ADD groups, respectively. Their median (IQR) age was 69 (64–75), 73 (65–77), and 74 (68–80) and median (IQR) years of education were 12 (6–16), 12 (6.5–16), and 9 (6–12), respectively. Their median (IQR) follow up months of the SCI, AMCI, and ADD groups were 79.3 (59.8–107.1), 47.1 (34.8–62.0), and 42.7 (29.8–59.2), respectively. Among 85 participants with SCI, 37(43.5%) converted to AMCI or AD. Also, 136 (56.7%) out of 240 AMCI patients converted to AD.

**Table 1:** Demographics.

	Lower-education (≤ 12)			Higher-education (> 12)			Total		
	SCI	AMCI	ADD	SCI	AMCI	ADD	SCI	AMCI	ADD
N	49	144	185	36	96	55	85	240	240
Number of visits, median (IQR)	5 (3–6)	4 (3–5)	4 (3–5)	4 (3–5)	4 (3–6)	4 (3–5)	4 (3–5)	4 (3–5)	4 (3–5)
Follow up month, median (IQR)	80.1 (59.8–113.2)	46.2 (34.1–60.9)	40.1 (29.3–58.8)	78.8 (61.8–92.8)	48.0 (35.2–65.1)	47.4 (35.1–65.6)	79.3 (59.8–107.1)	47.1 (34.8–62.0)	42.7 (29.8–59.2)
Age (year), median (IQR)	70 (66–75)	73 (64–77)	75 (69–80)	68 (60–74)	73 (67–78)	72 (65–76)	69 (64–75)	73 (65–77)	74 (68–80)
Male, no. (%)	3 (6.1)	28 (19.4)	39 (21.1)	16 (44.4)	65 (67.7)	35 (63.6)	19 (22)	93 (39)	75 (31)
Education (year), median (IQR)	8 (5–12)	9 (6–12)	6 (3–11)	16 (16–16)	16 (16–16)	16 (15–16)	12 (6–16)	12 (6.5–16)	9 (6–12)
Baseline CDR-SB, median (IQR)	0.5 (0.5–1.0)	1.5 (1.0–2.0)	4.0 (3.0–5.0)	0.5 (0–0.5)	1.0 (1.0–1.5)	3.5 (2.5–4.5)	0.5 (0.0–1.0)	1.5 (1.0–2.0)	4.0 (3.0–5.0)
APOE4 carries, no (%)^a^	10/31 (68)	50/120 (58)	54/110 (49)	13/26 (50)	35/84 (52)	19/31 (28)	23/57 (40)	85/204 (42)	73/141 (52)

ADD: Alzheimer’s disease dementia; AMCI: amnestic mild cognitive impairment; APOE4: apolipoprotein E4; CDR-SB: clinical dementia rating sum of boxes; SCI: subjective cognitive impairment; IQR: Inter-Quartile Range.

^a^APOE4 was analyzed in 402 patients. Participants with 1 or more copies of $\varepsilon$4 allele (i.e. $\varepsilon$2/4, $\varepsilon$3/4, $\varepsilon$4/4) are considered $\varepsilon$4 carriers.

### Development of a disease progression model of AD

**Figure 1:**
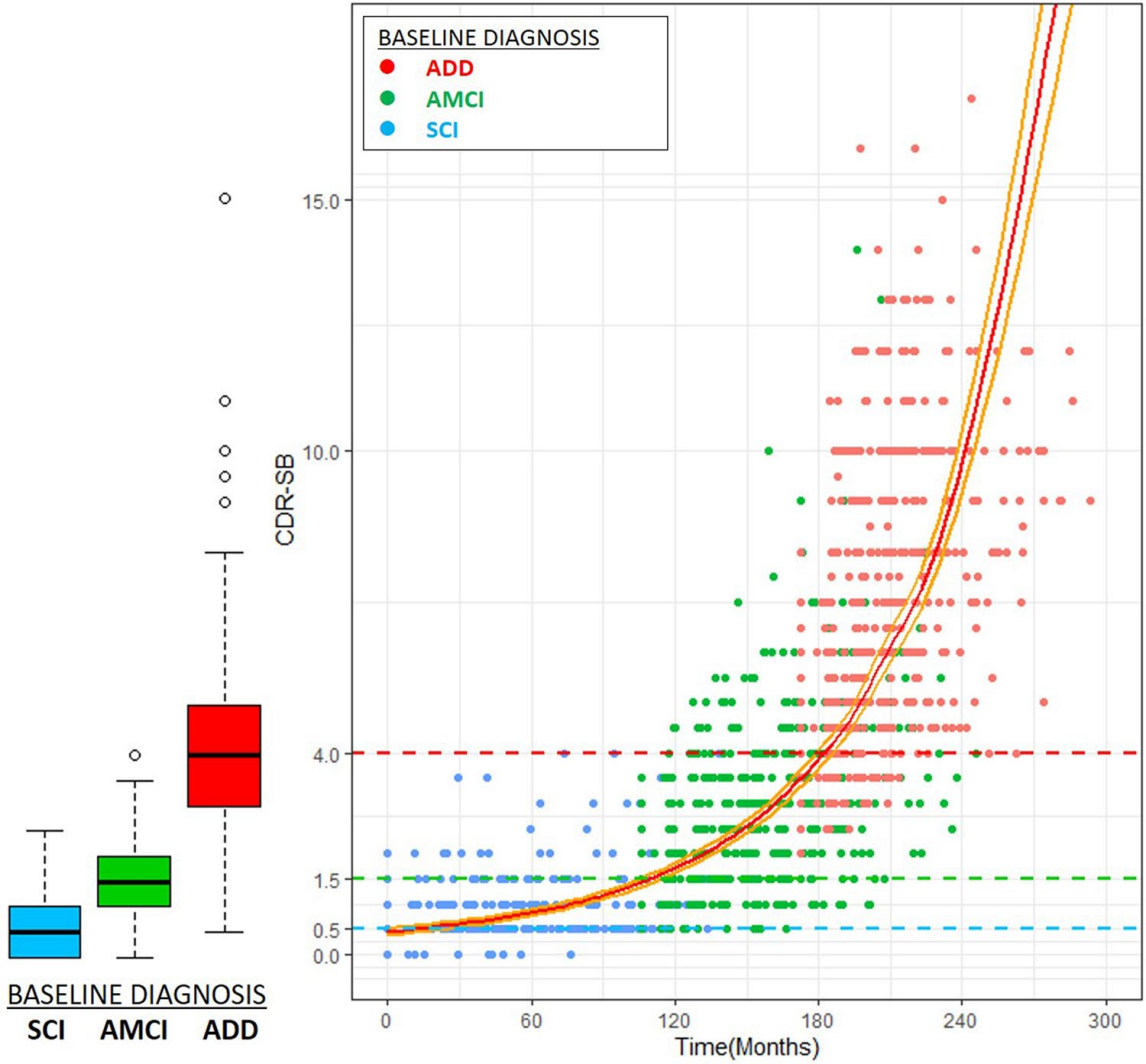
Disease progression model using CDR-SB. Each dot corresponds to a follow-up CDR-SB value. The color of the dot is determined according to its baseline diagnosis cohort: SCI (blue), AMCI (green), and ADD (red). The estimated model of CDR-SB from combined SCI, AMCI, and ADD cohorts shows that it takes 274.3 months to increase the CDR-SB value from 0 to 18 points. The predictive equation for the curve is as follows: $\text{ln}(\text{CDR-SB }+ 0.5) = -0.06008 + 0.004275 \times \text{ time }+ 0.000024 \times \text{ time}^{2}$. ADD: Alzheimer’s disease dementia; AMCI: amnestic mild cognitive impairment; CDR-SB: clinical dementia rating sum of boxes; SCI: subjective cognitive impairment.

Combining SCI, AMCI, and ADD cohorts, we developed a disease progression model of AD using clinical dementia rating sum of boxes (CDR-SB). Outliers were investigated using standardized residuals, and observations with absolute values of standardized residuals greater than 3 were considered as outliers. Akaike information criterion (AIC), Bayesian information criterion (BIC), and AIC with a correction for finite sample sizes (AICC) were used to evaluate the model fit before and after removing outliers, and it was identified that the model after removing outliers was better than the model before removing outliers (Supplementary table 1). The predictive equation for the curve was as follows: ln (CDR-SB + 0.5) = − 0.06008 + 0.004275 × time + 0.000024 × time^2^ (Fig. 1).

Based on the predictive equation, it takes 274.3 months to increase the CDR-SB score from 0 to 18 points. The predicted CDR-SB score of measurements to convert from the SCI to the AMCI group was 1.27 (95% CI 1.16–1.40) and the corresponding time was 116.5 months. The predicted CDR-SB value of measurements to convert from the AMCI to the ADD group was 3.95 (95% CI 3.74–4.19) and the corresponding time was 56.2 months (Table 2).

**Table 2:** Time to transition to disease status according to the level of education.

	Number of patients	Time to progression, months (95% CI)	
		SCI → AMCI	AMCI → ADD
Lower-education group (≤ 12 years)	378	105.8 (89.7, 122.0)	61.7 (57.6, 65.8)
Higher-education group (> 12 years)	187	141.8 (118.3, 165.2)	47.8 (41.6, 54.6)
Total	565	116.5 (103.6, 129.5)	56.2 (52.6, 60.5)

ADD: Alzheimer’s disease dementia; AMCI: amnestic mild cognitive impairment; SCI: subjective cognitive impairment.

### Development of a disease progression model of AD according to education level

**Figure 2:**
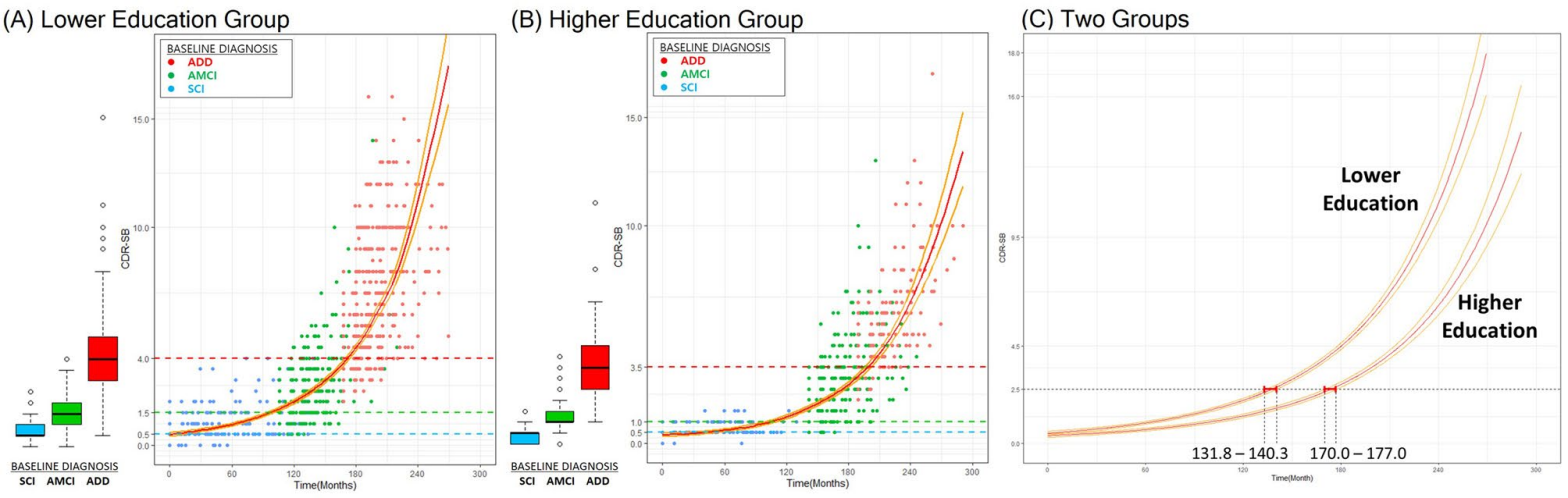
Disease progression model according to the level of education. Each dot indicates each follow-up CDR-SB value. The color of the dot is determined according to its baseline diagnosis cohort: SCI (blue), AMCI (green), and ADD (red). It takes less time to increase the CDR-SB value in the lower-education group in all sections. In the stages of SCI to AMCI, the CDR-SB value increases faster in the lower-education group. From the stage of AMCI to ADD, the CDR-SB value increases faster in the higher-education group. (**A**) Lower-education group. The estimated model in the lower-education group using CDR-SB. The predictive equation is: $\text{In}(\text{CDR SB}+0.5)=-0.01585+0.005247\times \text{time}+0.000021\times \text{time}\times {\text{time}}^{2}$. (**B**) Higher-education group. The estimated model in the higher-education group using CDR-SB. The predictive equation is: $\text{In}(\text{CDR SB}+0.5)=-0.1377+0.00323\times \text{time}+0.000022\times \text{time}\times {\text{time}}^{2}$. (**C**) Two groups. The lower-education group arrives faster than the higher-education group from CDR-SB 0–2.5. The two red bars indicate that the 95% CI (131.8, 140.3) of the lower-education group and the 95% CI (170.0, 177.0) of the higher-education group do not overlap at the time corresponding to CDR-SB 2.5. ADD: Alzheimer’s disease dementia; AMCI: amnestic mild cognitive impairment; CDR-SB: clinical dementia rating sum of boxes; CI: confidence interval; SCI: subjective cognitive impairment.

We also developed a disease progression model in lower- and higher-education groups. The predictive equations for the curves were as follows (Fig. 2):

$$\text{Lower-education group}{:}\; \text{In }(\text{CDR-SB }+ 0.5) = -0.01585 + 0.005247\times \text{time }+ 0.000021\times \text{time}^{2}$$

$$\text{Higher-education group}{:}\; \text{In }(\text{CDR-SB }+ 0.5) = -0.1377 + 0.00323\times \text{time }+ 0.000022\times \text{time}^{2}$$

Based on the predictive equations, the lower-education group takes 269.2 months to increase the CDR-SB score from 0 to 18 points. The predicted CDR-SB value for measurements to convert from the SCI to the AMCI group was 1.35 (95% CI 1.19–1.52) and the corresponding time was 105.8 months. The predicted CDR-SB value for measurements to convert from the AMCI to the ADD group was 4.04 (95% CI 3.78–4.31) and the corresponding time was 61.7 months.

The higher-education group takes 306.4 months to increase the CDR-SB value from 0 to 18 points. The predicted CDR-SB value for measurements to convert from the SCI to the AMCI group was 1.17 (95% CI 1.01–1.36) and the corresponding time was 141.8 months. The predicted CDR-SB value for measurements to convert from the AMCI to the ADD group was 3.68 (95% CI 3.31–4.09) and the corresponding time was 47.8 months.

We also observed the time it takes to increase the CDR-SB value in the model according to the level of education. In all sections, it takes less time to increase the CDR-SB value in the lower-education group (Table 3). As shown in Fig. 2C, the time corresponding to CDR-SB 2.5 was 137.1 month (95% CI 131.8, 140.3) in the lower-education group and 174.8 month (95% CI 170.0, 177.0) in the high education group. These results indicate that the 95% CI (131.8, 140.3) of the lower-education group and the 95% CI (170.0, 177.0) of the higher-education group do not overlap at the time corresponding to CDR-SB 2.5. Therefore, the lower-education group arrives significantly faster than the higher-education group at CDR-SB 2.5 from 0 point. The lower-education group reaches CDR-SB 2.5, 37.7 months ahead of the high education group. However, after CDR SB 2.5, there were no differences in the rate of change between the two groups.

**Table 3:** Time to CDR-SB increase according to level of education.

	Number of patients	Time to progression, months (95% CI)			
		CDR-SB 0 → 2.5	CDR-SB 2.5 → 4.5	CDR-SB 4.5 → 9.5	CDR-SB 9.5 → 16
Lower-education group (≤ 12 years)	378	137.1 (131.8, 140.3)	42.9 (35.8, 51.8)	50.0 (42.1, 58.8)	32.1 (21.8, 42.8)
Higher-education group (> 12 years)	187	174.8 (170.0, 177.1)	43.1 (36.1, 50.0)	49.8 (39.8, 60.3)	31.9 (15.8, 47.3)
Total	565	148.0 (145.2, 152.3)	41.3 (34.9, 48.3)	47.8 (41.3, 55.1)	30.6 (22.4, 39.4)

CDR-SB: clinical dementia rating sum of boxes.

We next performed a sensitivity analysis including non-decliners to examine if excluding non-decliners affected the estimation of the disease progress model. The disease progression model including non-decliners took longer (329.8 months) than our original model (274.3 months), which could be a predictable effect of non-decliner data (Supplementary figure 1). However, our original analysis produced a model that, when non-decliners were included, showed a consistent progression pattern where the lower-education group demonstrated a faster CDR-SB progression from SCI to AMCI than the higher-education group, and this trend disappeared from AMCI to ADD (Supplementary table 3, Supplementary figures 1 and 2).

## Discussion

We estimated the entire disease progression of AD in a model using CDR-SB follow-up data from three relatively large-sized cohorts, SCI, AMCI, and ADD. Based on the predictive equation, it takes 274.3 months to increase the CDR-SB value from 0 to 18 points. We also found that it takes 116.5 months to progress from SCI to AMCI and 56.2 months to progress from AMCI to ADD. In particular, the CDR-SB progression from SCI to AMCI was faster in the lower-education group compared to the higher-education group, and this trend disappeared from AMCI to ADD. Taken together, our findings suggest that it might be helpful to stage the current level of disease severity and establish management plans by education level.

In the present study, we developed a disease progression model for the AD spectrum. We found that the AD process takes 274.3 months (22.9 years) to progress from 0 to 18 points in the CDR-SB value. Previous studies were limited in showing the entire disease progression from SCI to the end stage of ADD. Instead, previous modeling studies have reported different disease progression rates depending on disease severity^10–12^, the prediction of disease onset time^13^, and the impact of biomarkers on the disease course^12,14–16^. In addition, our model is based on the CDR-SB, which is one of the most widely used dementia staging systems, and represents a validated, well-described, and reliable measure of disease progression^17,18^. The CDR-SB has the advantage of being able to measure changes precisely over time from SCI to the end stage of ADD. Thus, our disease progression model represents the patients’ functional ability across the whole disease spectrum.

Based on our predictive equation, it takes 116.5 months (9.7 years) to progress from SCI to AMCI and 56.2 months (4.7 years) to progress from AMCI to ADD. Similar to our results, one progression model study predicted that it took 7.9 years on average from cognitively normal to AMCI^19^. Another progression model study suggested that it took 4.3 years from AMCI to ADD^1^, and a previous study also showed that 60% of AMCI participants progress to ADD in 5 years^15^.

A noteworthy finding was that the lower-education group showed a faster progression from SCI to AMCI than the higher-education group, and this trend disappeared when progressing from AMCI to ADD. That is, many previous studies have shown that high education in cognitively normal participants has a protective effect on cognitive decline or development of dementia, whereas in the dementia stage, these protective effects disappeared or higher-educated dementia patients showed a steeper cognitive decline than lower-educated patients with dementia^20–24^. In contrast, other studies have reported that education level was not associated with the onset or rate of accelerated cognitive decline, but associated with differences in baseline cognition^15,23,25–27^. Inconsistencies in prior studies on the relationship between education level and cognitve decline might be influenced by baseline age, length of follow-up, and methodological or analytical factors^28^. However, our previous study showed that the protective effects in the higher-education group remain in the early stage of AMCI, whereas it disappeared in the late stage of AMCI^22^. Therefore, our findings of disappearance of protective effects of higher-education from AMCI to ADD stages might be related to the fact that the pathophysiological burden reaches a level severe enough not to be compensated by cognitive reserve during these periods^28^.

Interestingly, the CDR-SB value at the conversion of SCI to AMCI or AMCI to ADD is lower in the higher-education group than in the lower-education group. Perhaps this is because the higher-education group are more likely to be involved in cognitive tasks or occupational roles where subtle cognitive decline could be easily detected before the CDR-SB value progressed further. Alternatively, considering that CDR reflects the activity of daily living (ADL) as well as cognition, it might be reasonable to expect that ADL is relatively conserved even after cognitive decline in the higher-education group.

The principal strength of this study is that the disease progression model for the whole time using multiple single-cohorts was estimated with a well evaluated and large sample followed a maximum of 76 months. However, there are several limitations. First, the heuristic approach to modeling the whole disease spectrum using separate cohorts is a limitation of this study because of errors related to variables in the model. Although we showed that the range of the estimates of the converted time points was narrow, our model still has statistical methodology limitations^29–31^. Second, the CDR-SOB has not been administrated by an independent rater from other neuropsychological tests. To reduce the effect of this bias as much as possible, our CDR scores were evaluated by certified clinical psychologists and the patient's diagnosis was made by a multidisciplinary team composed of neuropsychologists and neurologists. In addition, our model might have a limitation in the early stages of AMCI when the ADL is not impaired. Third, other variables that often overlap with education level were not excluded. For instance, better social-economic states are more supportive of accomplishing a higher-education level, which may bring occupational attainment and good lifestyles^28^. Finally, the higher-education group is more self-aware of subjective cognitive impairment, which leads to earlier detection than what is found in the lower-education group. It might make the higher-education group appear to progress more slowly. If it is only for earlier detection of the higher-education group, the progress pattern should be the same in the early and late stage. However, this slow progression trend in the higher-education group disappeared when progressing from AMCI to ADD. It suggested that there was a protective effect of education in the early stage. Although there are several limitations, our findings are noteworthy because our model allows an estimate of the time course representing the disease trajectory using CDR-SB according to the level of education.

In conclusion, the developed model is suitable for describing the progression of disease in AD, which takes 274.3 months to advance from 0 to 18 points in the CDR-SB value. The effect of the level of education on disease progression differed according to disease severity. Our disease modeling provides us with more understanding of the effect of education on cognitive trajectories.

## Methods

### Participants

We enrolled 645 patients (129 SCI, 270 AMCI, and 246 ADD) who were followed up more than three times to obtain CDR-SB scores at the Samsung Medical Center from Jan. 2003 to Dec. 2015. Experienced neurologists evaluated the participants based on their clinical symptoms and reviews of medical/medication history, neuropsychological test results, magnetic resonance imaging (MRI), and laboratory tests. ADD patients met the criteria proposed by the National Institute of Neurological and Communicative Disorders and Stroke and the Alzheimer’s Disease and Related Disorders Association (NINCDS-ADRDA)^32^. The diagnosis of AMCI was based on the criteria proposed by Peterson and colleagues^33^ with inclusion of the following modifications: (1) Subjective cognitive complaints by the patient or his/her caregiver, (2) normal activities of daily living, (3) objective memory decline assessment below the 16th percentile on neuropsychological tests, and (4) absence of dementia. The SCI group were individuals who had a self-reported persistent decline in cognitive/memory capacity but were not impaired on neuropsychological tests^34^.

In all three groups, we excluded participants with other structural lesions such as territorial infarction, intracranial hemorrhage, brain tumor, hydrocephalus, or severe white matter hyperintensities (WMH) observed on brain MRI. Severe WMH on MRI was defined as a cap or a band ≥ 10 mm as well as a deep white matter lesion ≥ 25 mm as modified from the Fazekas ischemia criteria^35^. We also excluded 78 patients whose CDR-SB score decreased or did not change during follow up. Finally, we included 85 SCI, 240 AMCI, and 240 ADD patients. There was no significant difference in ADD risk (age, sex, education, APOE 4 carrier) between including decliner (included) and non-decliner (excluded) groups (Supplementary table 2).

### Standard protocol approvals, registrations, and patient consents

The Institutional Review Board at Samsung Medical Center approved our study and waived the need for informed consent since we used retrospective de-identified data collected during health exam visits. In addition, all methods were carried out in accordance with the approved guidelines.

### Education

We interviewed all patients and caregivers to evaluate detailed information about their educational background, including whether or not they had completed each step of education (elementary school, middle school, high school, college, and graduate school). We divided patients into two groups: those with more than 12 years of education and those with less. This cut-off was used because it corresponds to the duration of schooling that precedes the beginning of college in South Korea. Overall, 49 SCI, 144 AMCI, and 185 ADD patients were in the lower-education group (education ≤ 12 years) and 36 SCI, 96 AMCI, and 55 ADD patients were in the higher-education group (education > 12 years).

### Neuropsychological assessments

All patients underwent a standardized neuropsychological battery called the SNSB^36^ and the CDR-SB. The SNSB, which is performed in most memory clinics in South Korea, consists of tests for verbal and visual memory, attention, language, praxis, four elements of the Gerstmann syndrome, visuoconstructive function, frontal executive function, and the mini-mental state examination (MMSE). The CDR-SB is a three-point scale used to characterize six domains of cognitive and functional performance. CDR-SB scores from 0 to 18 were used.

To perform the SNSB and to rate CDR in a memory clinic at a university hospital, the certificate of a ‘Clinical Psychologist’ authorized by the Korean Clinical Psychological Association is required. The qualifications required to obtain a certificate are: A graduate with a master’s degree (clinical psychology major) who has completed three years of training under the guidance of a clinical psychologist or a graduate with a doctorate (clinical psychology major) who has completed two years of training under the guidance of a clinical psychologist. At least 3000 hours of training is required for three years at the mandatory training institute.

### Brain MRI scans

All patients underwent brain MRI including T2, FLAIR, T2* GRE, and three-dimensional (3D) T1 images at Samsung Medical Center using the same model of 3.0T MRI scanner (Philips 3.0T Achieva; Best, the Netherlands).

### Statistical analysis

**Figure 3:**
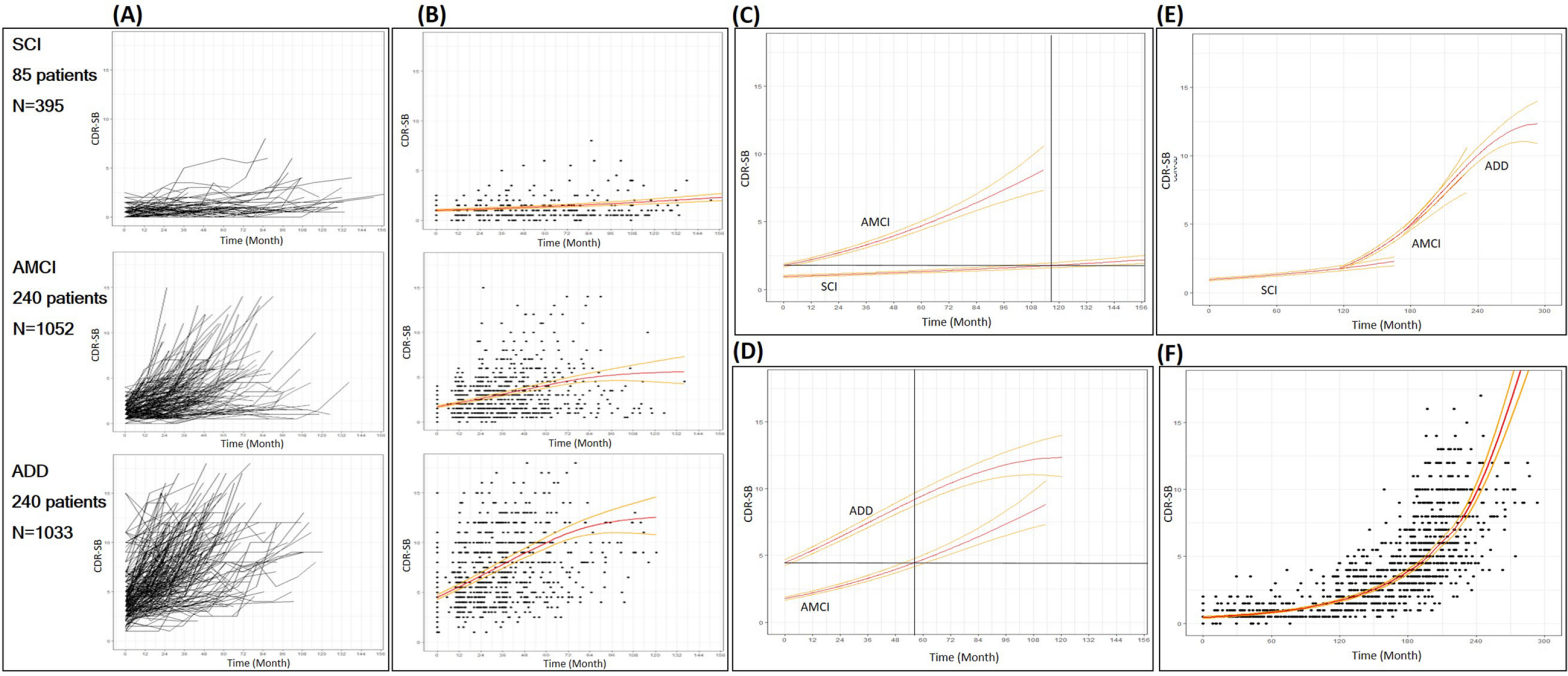
Disease progression modeling of CDR-SB in Alzheimer’s disease. (**A**) The pattern of CDR-SB values longitudinally observed in each of the three groups. (**B**) The smoothed line and the scatter plot with transformed CDR-SB using natural log in each of the three groups. (**C**) The estimated CDR-SB and the corresponding time for SCI and AMCI cohort to start to overlap. (**D**) The estimated CDR-SB and the corresponding time for AMCI and ADD cohort to start to overlap. (**E**) The AMCI cohort shifted by the time of 116.5 months and the ADD cohort shifted by 172.7 (= 116.5 + 56.2) months. (**F**) A disease progression model from SCI to the end stage of ADD. ADD: Alzheimer’s disease dementia; AMCI: amnestic mild cognitive impairment; CDR-SB: clinical dementia rating sum of boxes; SCI: subjective cognitive impairment.

The descriptive statistics for continuous variables are presented by median and inter-quartile range (IQR, 1st quartile, 3rd quartile) due to the non-normality of variables (Table 1). The Shapiro Wilk test was used to test the normality. Categorical variables were summarized by frequencies and percentages. The pattern of CDR-SB scores was examined using a spaghetti plot (Fig. 3A).

The entire disease continuum model was developed using the following three processes: (1) modelling for each disease cohort, (2) calculating the time for CDR-SB of the two consecutive disease cohorts to start to overlap, (3) constructing an entire disease continuum model. First, for the estimation of the model for longitudinal data, the mixed-effect model with a random effect for the patient and a fixed effect for the time was applied to each set of disease cohort data. CDR-SB with skewed distribution was transformed by the natural log after adding 0.5 to all scores because zero scores occur in CDR-SB. Time effect was fitted using a linear term or a quadratic term in each model after testing the significance of the quadratic term. For the diagnosis of the estimated model, studentized residuals were used to examine model assumptions and to detect outliers for each model. The observations with absolute studentized residuals greater than 3 were considered outliers and the model was re-estimated after excluding outliers from the data (Fig. 3B). The improvements in the goodness of fits to the model after excluding outliers were evaluated using Akaike information criterion (AIC), Bayesian information criterion (BIC), and AIC with a correction for finite sample size (AICC). Second, using the estimated model of each cohort, the point estimates and 95% confidence intervals (CI) of CDR-SB at the time measured for each patient were calculated. If a point estimate of CDR-SB in AMCI fell within 95% CIs of CDR-SB in SCI, this point estimate was considered as an overlapped CDR-SB between the two cohorts, SCI and AMCI. We found the smallest score among the overlapped CDR-SB in the AMCI cohort and substituted this score into the estimated model of the SCI cohort to calculate the corresponding time to this CDR-SB (Fig. 3C). Then, we shifted the data of the AMCI cohort to start from this time (Fig. 3E). Also, the same procedure was performed for the two consecutive AMCI and ADD cohort data points. (Fig. 3D,E). Finally, we constructed a single model using whole data from the three cohorts using a mixed-effect model. (Fig. 3F). To examine the effect of education level on disease progression, another disease progression model of the entire ADD continuum was developed in lower- and higher-education groups using mixed effect models. The time from SCI to AMCI and the time from AMCI to ADD were also calculated with this model. For all tests, a *p*-value < 0.05 was considered statistically significant. Statistical analysis was performed using SAS 9.4 (SAS Institute Inc, Cary, NC) and R 3.5.1 (Vienna, Austria) ggplot2 package.

## Supplementary information

Supplementary Information.

## Data Availability

The datasets generated during and/or analysed during the current study are available from the corresponding author on reasonable request.
