# Supplementary material for: Disease progression modeling of Alzheimer’s disease according to education level
Source: Sci Rep. 2020 Oct 8;10:16808. doi: 10.1038/s41598-020-73911-6 (PMC7544693; doi:10.1038/s41598-020-73911-6)
Supplement: Supplementary file 1 — Supplementary Information. [file 41598_2020_73911_MOESM1_ESM.doc]

# Disease progression modeling of Alzheimer’s disease

# according to education level

Ko Woon Kim^1,2,3,4#^, Sook Young Woo^5#^, Seonwoo Kim^5^, Hyemin Jang^1,6^, Yeshin Kim^1,7^, Soo Hyun Cho^1,8^, Si Eun Kim^1,9^, Seung Joo Kim^1,10^, Byoung-Soo Shin^2,3,4^, Hee Jin Kim^1,6^, Duk L. Na^1,6,11,12^, and Sang Won Seo^1,6,11,12,13,14^*

**Authors’ affiliations:**

^1^Department of Neurology, Samsung Medical Center, Sungkyunkwan University School of Medicine, Seoul, Korea

^2^Department of Neurology, School of Medicine, Jeonbuk National University Hospital, Jeonju, Korea

^3^Research Institute of Clinical Medicine of Jeonbuk National University, Jeonju, Korea

^4^Biomedical Institute of Jeonbuk National University Hospital, Jeonju, Korea

^5^Statistics and Data Center, Samsung Medical Center, Seoul, Korea

^6^Neuroscience Center, Samsung Medical Center, Seoul, Korea

^7^Department of Neurology, Kangwon National University Hospital, Kangwon National University College of Medicine, Chuncheon, Korea

^8^Department of Neurology, Chonnam National University Hospital, Gwangju, Korea

^9^Department of Neurology, Inje University College of Medicine, Haeundae Paik Hospital, Busan, Korea

^10^Department of Neurology, Gyeongsang National University School of Medicine and Gyeongsang National University Changwon Hospital, Changwon, Republic of Korea

^11^Department of Health Sciences and Technology, SAIHST, Sungkyunkwan University, Seoul, Korea

^12^Samsung Alzheimer Research Center, Samsung Medical Center, Seoul, Korea

^13^Department of Clinical Research Design and Evaluation, SAIHST, Sungkyunkwan University, Seoul, Korea

^14^Center for Clinical Epidemiology, Samsung Medical Center, Seoul, Korea

**^#^** **These authors contributed equally to this work.**

***Corresponding author**

**Sang Won Seo, MD, PhD**

Department of Neurology, Samsung Medical Center, Sungkyunkwan University School of Medicine, 81 Irwon-dong, Gangnam-gu, Seoul, 06351, Korea.

Tel.: +82-2-3410-1233/-3599, Fax: +82-2-3410-0052, E-mail: [sangwonseo@empal.com](mailto:sangwonseo@empal.com)

**Supplementary Tables**

**Table S1. Goodness of fit for the model without outliers, and with outliers**

|  |  | Before excluding outliers | After excluding outliers |
| --- | --- | --- | --- |
| SCI | AIC | 565.5 | 549.8 |
|  | AICC | 566.6 | 549.8 |
|  | BIC | 571.4 | 554.7 |
| AMCI | AIC | 1495.4 | 1393.7 |
|  | AICC | 1495.4 | 1393.7 |
|  | BIC | 1502.5 | 1400.6 |
| ADD | AIC | 845.4 | 672.2 |
|  | AICC | 845.4 | 672.2 |
|  | BIC | 852.4 | 679.1 |

AIC, Akaike information criterion; AICC, AIC with correction for finite sample size; BIC, Bayesian information criterion.

**Table S2. ADD risk between decliner (included) and non-decliner (excluded) groups**

|  | Decliner (N=565) | | | Non-decliner (N=78) | | | P value†  (Decliner vs. Non-decliner) | | |
| --- | --- | --- | --- | --- | --- | --- | --- | --- | --- |
|  | SCI | AMCI | ADD | SCI | AMCI | ADD | SCI | AMCI | ADD |
| N | 85 | 240 | 240 | 44 | 30 | 4 | - | - | - |
| Age (year), median (IQR) | 69 (64,75) | 73 (65,77) | 74 (68,80) | 68 (64, 73) | 71 (64, 77) | 73 (71, 75) | 0.521 | 0.426 | 0.732 |
| Male, no. (%) | 19 (22) | 93 (39) | 75 (31) | 15 (34) | 11 (37) | 0 (0) | 0.141 | 0.825 | 0.183 |
| Education (year), median (IQR) | 12 (6,16) | 12 (6.5, 16) | 9 (6, 12) | 12 (9, 16) | 12 (9, 16) | 11 (8, 14) | 0.587 | 0.953 | 0.324 |
| APOE4 carriers (%)‡ | 23/57 (40) | 85/204 (42) | 73/141 (52) | 6/30 (20) | 7/29 (24) | 1/3 (33) | 0.056 | 0.071 | 0.527 |

ADD: Alzheimer’s disease dementia; AMCI: amnestic mild cognitive impairment; APOE4: apolipoprotein E4; CDR-SB: clinical dementia rating sum of boxes; SCI: subjective cognitive impairment

IQR: Inter-Quartile Range

†Mann-Whitney test for continuous variables. Chi-square test for categorical variables

‡ APOE4 was analyzed in 402 patients. Participants with 1 or more copies of
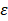
4 allele (i.e.
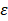
2/4,
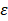
3/4,


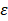
4/4) are considered
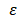
4 carriers.

**Table S3. Time to transition to disease status according to the level of education between decliner (included) and non-decliner (excluded) groups**

|  | Time to progression (months) | | | |
| --- | --- | --- | --- | --- |
|  | Lower-education group  (≤ 12years) | | Higher-education group  (> 12years) | |
|  | SCI → AMCI | AMCI → ADD | SCI → AMCI | AMCI → ADD |
| Excluding non-decliner (N=565) | 105.8 | 61.7 | 141.8 | 47.8 |
| Including non-decliner (N=643) | 137.3 | 95.3 | 145.7 | 62.2 |

ADD: Alzheimer’s disease dementia; AMCI: amnestic mild cognitive impairment; SCI: subjective cognitive impairment

**Supplementary Figures**


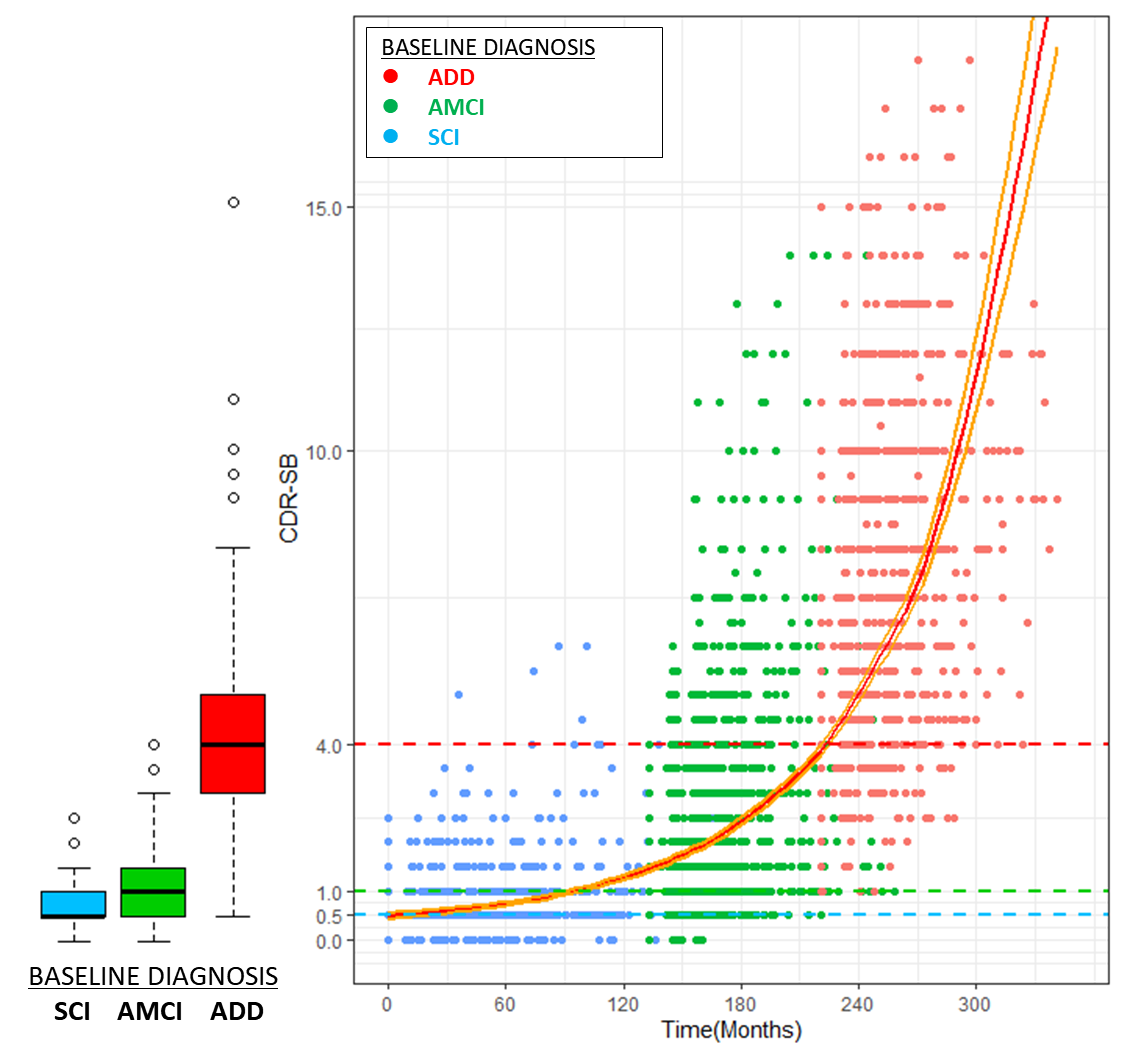


**Figure S1.** **Disease progression model using CDR-SB data including non-decliners**

Each dot indicates each follow-up CDR-SB value. The color of the dot is determined according to its baseline diagnosis cohort: SCI (blue), AMCI (green), and ADD (red). The predictive equation for the curve is as follows: ln (CDR-SB + 0.5) = -0.01708 + 0.002634 × time + 0.000019 × time^2^.

The predicted CDR-SB value for measurements to convert from the SCI to the AMCI group was 1.16 (95% CI: 1.05–1.28) and the corresponding time was 133.5 months. The predicted CDR-SB value for measurements to convert from the AMCI to the ADD group was 4.04 (95% CI: 3.81–4.29) and the corresponding time was 87.3 months.

Abbreviations: ADD, Alzheimer’s disease dementia; AMCI, amnestic mild cognitive impairment; CDR-SB, clinical dementia rating sum of boxes; SCI, subjective cognitive impairment.


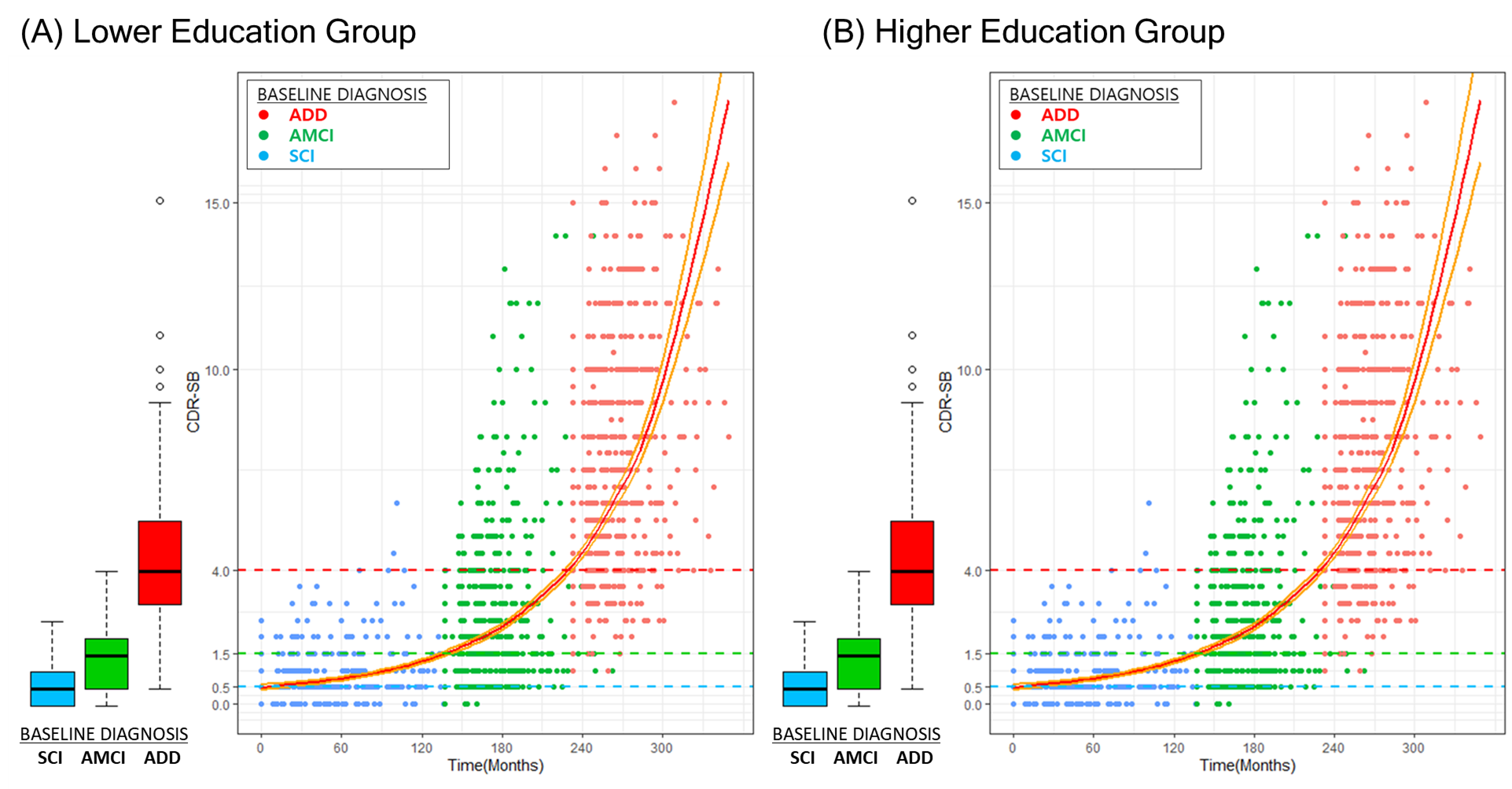


**Figure S2. Disease progression model according to the level of education**

Each dot indicates each follow-up CDR-SB values. The color of the dot is determined according to its baseline diagnosis cohort: SCI (blue), AMCI (green), and ADD (red). Our original analysis produced a model that, when non-decliners were included, showed a consistent progression pattern where the lower-education group demonstrated a faster CDR-SB progression from SCI to AMCI than the higher-education group, and this trend disappeared from AMCI to ADD

(A) The estimated model in the lower-education group using CDR-SB. The predictive equation is: In (CDR-SB + 0.5) = - 0.00821 + 0.002911×time + 0.000016×time^2^.

The predicted CDR-SB value for measurements to convert from the SCI to the AMCI group was 1.23 (95% CI: 1.08–1.39) and the corresponding time was 137.3 months (105.8M). The predicted CDR-SB value for measurements to convert from the AMCI to the ADD group was 4.2 (95% CI: 3.91–4.5) and the corresponding time was 95.3 months.

(B) The estimated model in the higher-education group using CDR-SB. The predictive equation is: In (CDR-SB + 0.5) = 0.01417 - 0.00009×time + 0.00003×time^2^.

The predicted CDR-SB value for measurements to convert from the SCI to the AMCI group was 1.08 (95% CI: 0.91–1.26) and the corresponding time was 145.7 months. The predicted CDR-SB value for measurements to convert from the AMCI to the ADD group was 3.52 (95% CI: 3.13–3.96) and the corresponding time was 62.2 months.

ADD: Alzheimer’s disease dementia; AMCI:amnestic mild cognitive impairment; CDR-SB: clinical dementia rating sum of boxes; SCI: subjective cognitive impairment.
